# Post-treatment levels of plasma 25- and 1,25-dihydroxy vitamin D and mortality in men with aggressive prostate cancer

**DOI:** 10.1038/s41598-020-62182-w

**Published:** 2020-05-08

**Authors:** Visalini Nair-Shalliker, Albert Bang, Sam Egger, Mark Clements, Robert A. Gardiner, Anne Kricker, Markus J. Seibel, Suzanne K. Chambers, Michael G. Kimlin, Bruce K. Armstrong, David P. Smith

**Affiliations:** 1 Cancer Research Division, Cancer Council NSW, Sydney, NSW Australia; 2 Sydney School of Public Health, The University of Sydney, Sydney, NSW Australia; 3 Department of Clinical Medicine, Macquarie University, Sydney, Australia; 4 Karolinka Institute, Stockholm, Sweden; 5 University of Queensland, Brisbane, Australia; 6 Bone Research Program, ANZAC Research Institute, and Concord Medical School, The University of Sydney, Sydney, Australia; 7 Faculty of Management, University Technology of Sydney, Sydney, Australia; 8 Health Research Institute, University of the Sunshine Coast, Queensland, Australia; 9 School of Population Health, University of Western Australia, Perth, Western Australia Australia

**Keywords:** Risk factors, Urology

## Abstract

Vitamin D may reduce mortality from prostate cancer (PC). We examined the associations of post-treatment plasma 25-hydroxyvitamin D and 1,25-dihydroxyvitamin D concentrations with PC mortality. Participants were PC cases from the New South Wales Prostate Cancer Care. All contactable and consenting participants, at 4.9 to 8.6 years after diagnosis, were interviewed and had plasma 25-hydroxyvitamin D (25(OH)D) and 1,25-dihydroxyvitamin D (1,25(OH)_2_D) measured in blood specimens. Cox regression allowing for left-truncation was used to calculate adjusted mortality hazards ratios (HR) and 95% confidence intervals (95% CI) for all-cause and PC-specific mortality in relation to vitamin D levels and other potentially-predictive variables. Of the participants (n = 111; 75·9% response rate), there were 198 deaths from any cause and 41 from PC in the study period. Plasma 25(OH)D was not associated with all-cause or PC-specific mortality (p-values > 0·10). Plasma 1,25(OH)_2_D was inversely associated with all-cause mortality (HR for highest relative to lowest quartile = 0·45; 95% CI: 0·29–0·69), and PC-specific mortality (HR = 0·40; 95% CI: 0·14–1·19). These associations were apparent only in men with aggressive PC: all-cause mortality HR = 0·28 (95% CI·0·15–0·52; p-interaction = 0·07) and PC-specific mortality HR = 0·26 (95% CI: 0·07–1.00). Time spent outdoors was also associated with lower all-cause (HR for 4^th^ relative to 1^st^ exposure quartile = 0·42; 95% CI: 0·24–0·75) and PC-specific (HR = 0·48; 95% CI: 0·14–1·64) mortality, although the 95% CI for the latter was wide. The inverse association between post-treatment plasma 1,25(OH)_2_D levels and all-cause and PC-specific mortality in men with aggressive PC, suggest a possible beneficial effect of vitamin D supplementation in these men.

## Introduction

Prostate cancer (PC) is the most commonly diagnosed cancer and the third most common cause of cancer death in men in developed countries^1^. Although the five-year survival rate for prostate cancer is ~95%, it is the second most common cause of death in Australian men. It is estimated that in 2018, 1 in 31 Australian men will die from prostate cancer by their 85^th^ birthday^2^. Thus, identifying modifiable factors that may improve a man’s survival after a prostate cancer diagnosis is important. There is some evidence that vitamin D may influence prostate cancer development and progression, and thus may be such a factor^3^.

The two main metabolites of vitamin D are the pro-hormone calcidiol (25(OH)D) which is converted to the biologically active hormone calcitriol (1,25(OH)_2_D) in a tightly regulated process. 1,25(OH)_2_D has anti-proliferative, pro-differentiating and pro-apoptotic properties on a range of cells and tissues, including cancers. The pro-hormone, 25(OH)D, is stable and consequently more abundant in serum than 1,25(OH)_2_D and is thus used to infer vitamin D status. A circulating level of 25(OH)D above 50 nmol/L is generally considered sufficient while that for 1,25(OH)_2_D is not defined^4^.

High circulating 25(OH)D levels may improve PC survival^5^. Long term follow-up of men from the Health Professional Follow-up (HPFS) and Physicians Health (PHS) studies showed that men with pre-diagnosis circulating 25(OH)D below 40nmol/L had higher PC-specific mortality than those with levels above 90nmol/L (HR = 1·59; 95% CI: 1·06, 2·39)^6–9^. This association was more evident in men with aggressive disease^6,7^. A small Norwegian study comparing blood specimens collected pre- and post-treatment showed a stronger association of post-treatment than pre-treatment vitamin D levels with outcome^10^, thus suggesting that post-diagnosis vitamin D status may be a better predictor of PC outcome than pre-diagnosis status.

Risks of both prostate cancer and vitamin D deficiency (<30 nmol/L) increase with advancing age. The development of metastases, which are usually in bone, is led by the intercellular communication between PC cells and their microenvironment^11^. Studies in prostate cancer cell lines showed that vitamin D deficiency stimulates prostate cancer growth in bone and have attributed this to possible changes in the bone microenvironment or through direct actions of the unliganded vitamin D receptor^12^. Two clinical trials in PC cases showed that although vitamin D supplementation increased circulating 25(OH)D and 1,25(OH)_2_D levels in blood and tissue, reduced proliferation in prostate cancer cells was only associated with increased 1,25(OH)_2_D levels^13,14^. These results suggest that (i) high 25(OH)D levels may induce intracrine activity in the prostate and reduce proliferation, and thus maintaining these high levels may prevent PC progression and improve survival, and (ii) that blood 1,25(OH)_2_D levels are a predictors of PC outcome. To fully understand the relationship between vitamin D and PC outcomes, the effects of both 25(OH)D and 1,25(OH)_2_D levels need to be analysed.

The primary objective of the current study was to examine the associations of plasma vitamin levels of 25(OH)D and 1,25(OH)_2_D, after PC treatment with death from all-causes and death from PC in an Australian cohort of PC patients^15^. We also explored the associations of other factors previously shown to be associated with circulating vitamin D levels with PC mortality. We hypothesised a positive association between circulating vitamin D levels and PC survival.

## Materials and Method

### Study population

Participants in the New South Wales (NSW) Prostate Cancer Care and Outcome Study (PCOS) were eligible to participate in the present study (PCOSun).

PCOS participants were residents of NSW identified through the NSW Cancer Registry as diagnosed with pathologically proven PC when aged 70 years or less between September 2000 and October 2002· Of these men, 62% (n = 1,995) participated in PCOS^15^. PCOS participants were interviewed first at three months after PC diagnosis and reinvited for quality of life assessments at one, two, three, five, ten and fifteen years later. All PCOS participants who remained contactable and had not actively withdrawn from PCOS by 2007 (n = 1572) were invited by letter to participate in PCOSun. Details of the study protocol have been published^15^.

Each man who accepted our invitation and gave written informed consent, was asked to complete an interview and given a request form to take to their nearest pathology specimen collection centre for collection of blood for measurement of plasma 25(OH)D and 1,25(OH)_2_D.

The Cancer Council NSW Human Research Ethics Committee (#217) approved this study.

### Interview

The computer assisted telephone interview included questions on ethnicity, skin pigmentation, tanability, diet, vitamin D supplementation, and time spent outdoors between 9 am and 5 pm on weekends and weekdays or days-off in the 4 weeks before blood collection.

### Plasma vitamin D analysis

Participants’ blood specimens were collected into EDTA tubes and processed within 48 hours of collection. All plasma samples were aliquoted and stored at −80 °C and analysed for 25(OH)D and 1,25(OH)_2_D at the end of sample collection. Personnel at RDDT Laboratories (vivo Pharm Co. RMIT University, Melbourne, Australia) who were blinded to participant status performed all vitamin D analyses, according to their standard operating procedures (SOP). Analyses of 25(OH)D were carried out by high-pressure liquid chromatography-mass spectrometry (API 4000 QTRAP LC-MS/MS), which, according to RDDT’s protocol, has a sensitivity of 6·9 nmol/L for 25(OH)D_3_. All analyses were based on at least a 6-point standard calibration curve with a standard acceptance criterion of <20% for any given standard, with an intra-assay precision of <20% and an intra-assay accuracy of ±15%. Plasma 1,25(OH)_2_D was determined in duplicate using chemiluminescence immunoassay (IDS-iSYS,EIA Immuno Diagnostics Systems). The assay has a sensitivity of 6 pmol/L, and an intra-assay precision of <20%. The 1,25(OH)_2_D assay measured the dihydroxylated forms of both vitamin D_2_ and vitamin D_3·_

### Study endpoints

Participants’ personal details were linked probabilistically to the Australian National Death Index (compiled from death registration records) to determine vital status and, if dead, the cause of death. Linkage was complete to December 2015 for PCOS. Data were obtained on fact, date and registered cause of death.

### Data analysis

Variables used in the analysis are detailed in Table 1· The residential Accessibility and Remoteness Index of Australia (ARIA plus), was derived from residential postcode, and used to classify each participant’s place of residence^16^. The Index of Relative Socioeconomic Disadvantage was used as a measure of local government area socioeconomic status (SES), and ranked into population quintiles, with the lowest quintile the least disadvantaged^17^. Body Mass Index (BMI), based on self-reported measures of weight and height before PC diagnosis, was calculated as weight (kg)/[height (m)]^2^ and classified into the World Health Organisation’s standard groups. The few (n = 5) in the underweight category (<18·5 kg/m^2^) were combined with those in the “normal” BMI group (18·5–24·9 kg/m^2^). Season of blood collection was defined as “Summer”, “Autumn”, “Winter” and “Spring” for bloods drawn between December and February, March and May, June and August, and September and November respectively.

Co-morbidity was categorised as having no self-reported co-morbid condition from the following list or having one or more of these conditions: arthritis, diabetes, heart disease, stroke, high blood pressure, depression or anxiety, inflammatory bowel disease, stomach ulcers, asthma, angina or liver disease.

Men were classified as having aggressive or non-aggressive PC at diagnosis, according to the National Comprehensive Cancer Network criteria^18^, which classify clinically significant PC as aggressive if the Gleason score is >7, clinical stage is T2b or greater, total PSA levels are >10 ng/mL, or a regional lymph node or distant metastasis was found at diagnosis.

Weekly sun exposure hours were estimated by multiplying hours reported per weekday by five, and hours on weekend days or days off by two and summing them. Participants were ranked according to their hours of sun exposure and divided into quartiles for analysis; the lowest sun exposure quartile was used as the reference category. Plasma 25(OH)D and 1,25(OH)_2_D levels were divided into quartiles, using the lowest category in each as the reference group. All quartiles were relative to the PCOSun population.

Men with missing data for covariates were excluded from analyses that required them.

### Statistical analysis

Cox regression models allowing for left-truncation were used to calculate mortality hazards ratios (HR) and their 95% confidence intervals^19^. The underlying time scale for the Cox regression was time from diagnosis, with entry at the blood draw and exit at death, emigration or the end of follow-up, whichever came first. HRs were adjusted for age, place of residence, socioeconomic status, birth region, BMI, initial PC treatment, co-morbidity, PC aggressiveness and season of blood collection. We also examined, in separate Cox models, the associations of weekly outdoor exposure (estimator of UV exposure), skin colour and tanability with outcomes. The interactions of the main exposures of interest, which were plasma 25(OH)D and 1,25(OH)_2_D levels, sun exposure, ability to tan, and skin colour, with aggressiveness of disease, treatment and cohort (PCOS or ProsCan) were examined, and declared present if the *p*-value for interaction was <0·10. Nominal p-values are shown for each statistical test with no adjustments made for multiple comparisons. Frequencies in cells with less than 5 participants were not reported. All analyses were done using SAS software version 9·1.

Due to seasonal variation of vitamin D levels, season of blood collection is plausibly on a causal pathway between vitamin D and death, and thus a sensitivity analysis excluding season of blood collection from Cox models was conducted.

### Ethics approval

All procedures performed in studies involving human participants were in accordance with the ethical standards of the institutional and national research committee - The Cancer Council NSW Human Research Ethics Committee (#217).

### Informed consent

Informed consent was obtained from all individual participants included in the study.

## Results

**Figure 1:**
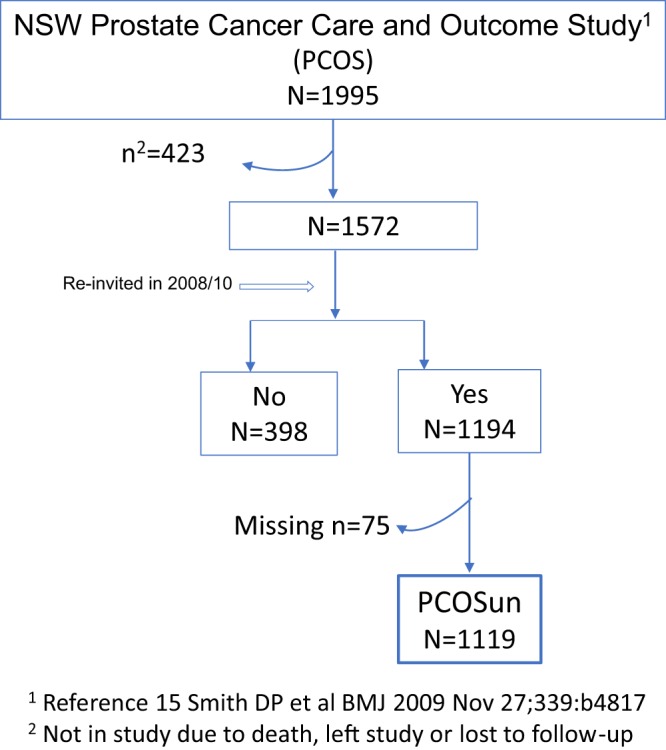
Flow diagram showing final derivation of participants in the PCOSun cohort.

Of the eligible and consenting PCOS participants (n = 1194; participation rate 75.9%) 75 were excluded from the analysis because of missing data, leaving 1119 (86·7% of eligible and consenting) men in the final analysis (Fig. 1).

Median age at blood collection was 68 years (range 49 to 77 years), and median follow-up time after blood collection was 97 months. There were 198 deaths due to any cause during the study period of which 41 were certified to PC.

Plasma 25(OH)D and 1,25(OH)_2_D levels were positively correlated (Pearson correlation = 0.28; p-value < 0.0001). Mean plasma 25(OH)D and 1,25(OH)_2_D levels were 66.0 nmol/L and 90.9 pmol/L, respectively (Table 1). Plasma 25(OH)D levels were higher in men who were born in Australia, were diagnosed with non-aggressive disease, received radiotherapy as primary treatment and had blood drawn in summer (Table 1; p-value < 0.05). They were also positively associated with increasing remoteness of residence, increasing time spent outdoors, and increasing ability to tan. Plasma 1,25(OH)_2_D levels (Table 1) were higher in men who were diagnosed with non-aggressive disease, received radiotherapy as primary treatment, had no comorbidities, and had blood drawn in autumn/winter (p-value < 0.05) and were economically advantaged. They were inversely associated with age and BMI. Vitamin D was not included in this analysis as only 2.4% of participants reported taking them.

There was no consistent evidence that plasma 25(OH)D was associated with all-cause or prostate-specific mortality (p-value > 0·1; Table 2). Plasma 1,25(OH)_2_D was significantly associated with all-cause mortality (HR for highest relative to lowest quartile = 0·45, 95% CI 0·29, 0·69; p = 0·0047; Table 2). While there was a similar trend for PC specific mortality (HR 0·40, 95% CI 0·14, 1·19; p = 0·26) the p-value was high. Weekly hours of outdoor exposure were also inversely associated with all-cause mortality (HR for highest relative to lowest quartile was 0·55, 95% CI: 0·36, 0·84; p = 0·053; Table 2). Adjustment for plasma 1,25(OH)_2_D did not appreciably change this inverse association. Skin colour and ability to tan were not associated with all-cause or PC-specific mortality.

**Table 1:** Characteristics, mean plasma vitamin D levels and mortality outcomes of PCOSun^*^ participants with complete data for analysis.

Characteristics	Participants	25 OHD (SD)	1,25(OH)_2_D (SD)	Deaths (n)	
	n (%)	(nmol/L)	(pmol/L)	Total	PC
All participants	1119 (100.0)	66.0 (21.3)	90.9 (34.7)	198	41
**Region of birth**					
Australia	872 (77.9)	66.7 (21.0)	91.2 (35.0)	158	29
Other	247 (22.1)	63.5 (22.3)	89.8 (33.4)	40	12
P heterogeneity		0.039	0.57		
**Prostate cancer grade at diagnosis**					
Non-aggressive	528 (47.2)	68.6 (21.3)	93.7 (35.6)	82	11
Aggressive	591 (52.8)	63.7 (21.1)	88.4 (33.6)	116	30
P heterogeneity		0.0001	0.011		
**Primary Prostate cancer treatment**					
Active Surveillance^†^	120 (10.7)	65.6 (19.5)	85.1 (31.3)	26	NR^‡^
Radical prostatectomy	699 (62.5)	66.7 (20.9)	93.2 (34.0)	90	21
Low-dose brachytherapy	39 (3.5)	71.1 (19.3)	110.8 (39.7)	5	NR^‡^
External beam radiotherapy	65 (5.8)	67.9 (26.8)	93.7 (40.5)	13	NR^‡^
Androgen deprivation therapy^††^	196 (17.5)	62.2 (22.0)	81.4 (32.9)	64	17
P heterogeneity		0.044	<0.0001		
**Comorbidities**					
None	435 (38.9)	66.1 (20.9)	96.7 (35.5)	65	14
1 or more	684 (61.1)	66.0 (21.6)	87.2 (33.6)	133	27
P heterogeneity		0.94	<0.0001		
**Season of blood draw**					
Spring	818 (73.1)	62.6 (19.9)	88.8 (34.7)	138	28
Summer	261 (23.3)	75.8 (22.5)	96.7 (34.2)	55	NR^‡^
Autumn & Winter	40 (3.6)	72.0 (20.2)	97.0 (33.6)	5	NR^‡^
p-value <0.00010.003 P heterogeneity		<0.0001	0.003		
**Age at diagnosis (years)**					
49–54	152 (13.6)	69.8 (22.4)	101.0 (33.2)	15	5
55–59	271 (24.2)	66.2 (22.1)	93.7 (35.8)	34	9
60–64	319 (28.5)	65.2 (19.4)	87.9 (33.5)	47	14
65–85	377 (33.7)	65.1 (21.8)	87.3 (34.5)	102	13
P heterogeneity		0.11	<0.0001		
**Body Mass Index (BMI)**					
Underweight and normal weight	343 (30.7)	67.2 (22.8)	95.8 (37.2)	56	11
Overweight	555 (49.6)	66.3 (20.6)	90.5 (33.2)	94	19
Obese	221 (19.7)	63.3 (20.6)	84.5 (33.1)	48	11
P heterogeneity		0.092	0.00070		
**Socioeconomic status (SES)**					
1 (Most advantaged)	279 (24.9)	65.6 (20.9)	97.8 (37.0)	35	7
2	190 (17.0)	64.0 (20.6)	89.6 (34.7)	32	6
3	277 (24.8)	65.1 (20.4)	91.2 (33.7)	54	12
4	215 (19.2)	70.0 (23.6)	88.0 (33.2)	47	8
5 (least advantaged)	158 (14.1)	65.3 (20.8)	83.8 (32.1)	30	8
P heterogeneity		0.041	0.00060		
**Place of residence**					
Major cities	751 (67.1)	64.5 (20.9)	92.0 (35.7)	115	30
Inner regional	286 (25.6)	68.3 (21.2)	88.4 (32.4)	62	NR^‡^
Outer regional/Remote	82 (7.3)	72.4 (23.5)	89.5 (32.6)	21	NR^‡^
P heterogeneity		0.00070	0.30		
**Weekly hours of outdoor exposure**					
0–10·5 (inclusive)	255 (22.8)	59.562·7 (20.6)	87.2 (29.8)85·3	59	13
10·6–18.0	272 (24.3)	63.2 (20.4)66·5	94.2 (37.8)92·9	49	11
18.1–28.0	318 (28.4)	69.4 (20.0)71·5	92.6 (36.7)90·8	54	11
28.1–56·0	274 (24.5)	71.0 (22.4)73·6	89.1 (32.8)88·6	36	6
P heterogeneity		<0.0001	0.07		
**Skin colour**					
Very fair	161 (14.4)	64.0 (20.1)	90.0 (35.5)	NR^‡^	NR^‡^
Fair	637 (56.9)	65.9 (21.7)	90.0 (32.8)	113	23
Light olive	288 (25.7)	67.9 (21.0)	93.2 (38.2)	50	8
Dark olive/Brown/Black	33 (2.9)	62.0 (22.4)	92.6 (34.6)	NR^‡^	NR^‡^
P heterogeneity		0.17	0.61		
**Ability to tan**					
Deeply tanned	326 (29.1)	70.4 (23.5)	89.9 (35.4)	51	10
Moderately tanned	521 (46.6)	64.5 (20.6)	91.4 (34.6)	94	19
Mildly/no suntan	272 (24.3)	63.6 (19.1)	91.1 (34.0)	53	12
P heterogeneity		<0.0001	0.82		

^*^Follow up until December 2015 for NSW.

^†^Includes watchful waiting.

^††^Includes orchiectomy.

^‡^Not reported (NR) as sample size in one of these cells has less than 5 men.

**Table 2:** Adjusted mortality hazard ratios and 95% CI for deaths from all-causes and deaths from PC according to quartiles of plasma vitamin D and time spent outdoors, and categories of sun sensitivity in PCOSun participants (n = 1119).

Exposures	Person years	All-cause mortality		PC-specific mortality mortalityProstate cancer mortality	
		Deaths (n)	HR^†^(95% CI)	Deaths (n)	HR^†^(95% CI)
25 OHD (nmol/L)^*^					
14.23–53·82	2357.4	70	1·00	15	1·00
53·82–66·09	2238.2	48	0·74 (0·51, 1·07)	14	1·10 (0·52, 2·31)
66·09–80·25	2030.8	37	0·66 (0·44, 0·99)	5	0·43 (0·15, 1.20)
80·25–174.68	1754.5	43	0·79 (0·52, 1·19)	7	0·81 (0·31, 2·12)
P heterogeneity			0·19		0·33
**1,25(OH)**_**2**_D^*^(pmol/L)^*^					
4.84–67.24	1906.7	68	1·00	12	1·00
67.24–83·87	2085.2	49	0·76 (0·52, 1·10)	14	1·15 (0·52, 2·53)
83·87–106·56	2062.3	50	0·78 (0·54, 1·14)	10	0·84 (0·35, 2.01)
106·56–263.88	2326.6	31	0·45 (0·29, 0·69)	5	0·40 (0·14, 1·19)
P heterogeneity			0·0047		0·26
Weekly hours of outdoor exposure^*^					
0.1–10·5	1856.1	59	1·00	13	1·00
10·5–18.0	2048.3	49	0·78 (0·53, 1·15)	11	0·83 (0·37, 1·86)
18.1–28.0	2373.0	54	0·74 (0·51, 1·08)	11	0·76 (0·33, 1·73)
28.1–56·0	2103.4	36	0·55 (0·36, 0·84)	6	0·43 (0·16, 1.14)
P heterogeneity			0·053		0·40
**Skin colour**					
Very fair	1187.1	NR^††^	1·00	NR^††^	1·00
Fair	4771.5	113	0·92 (0·61, 1·36)	29	0·75 (0·33, 1·70)
Light olive	2171.9	50	0·93 (0·59, 1·47)	10	0·61 (0·23, 1·67)
Dark olive/Black	250.4	NR^††^	0·51 (0·15, 1·70)	NR^††^	1·12 (0·23, 5·49)
P heterogeneity			0·75		0·75
**Ability to tan**					
Deeply tanned	2452.1	51	0·82 (0·58 1·16)	10	0.89 (0·41, 1.93)
Moderately tanned	3926.7	94	1·00	19	1·00
Mildly/no tan	2002.1	53	1·11 (0·79, 1·56)	12	1·31 (0·63, 2·73)
P heterogeneity			0·32		0·65

^*^Categories are quartiles of exposure.

^**†**^Hazards ratio (HR) and 95% CI adjusted for study, age, place of residence, socioeconomic status, region of birth, BMI, PC treatment, PC grade at diagnosis, comorbidities, season of blood collection, and weekly hours of outdoor exposure.

^**††**^Not reported (NR) as sample size in one of these cells has less than 5 men.

The sensitivity analysis excluding season of blood collection from the regression did not appreciably change the size of the hazard ratios (results not shown).

Of the 30 interactions examined, only the p-value of that between 1,25(OH)_2_D levels and disease aggressiveness (p-interaction = 0·070) was relatively low. The association of 1,25(OH)_2_D with all-cause and PC-specific mortality appeared to be limited to men with aggressive disease (Table 3): all-cause mortality HR for highest relative to lowest quartile = 0·28 (95% CI: 0·15, 0·52) and corresponding PC-specific mortality HR = 0·26 (95% CI: 0·07, 1.00). There was no strong or consistent pattern for an association of ability to tan with all-cause mortality or modification of this association by disease aggressiveness.

**Table 3:** Adjusted mortality hazard ratios and 95% CI for deaths from all-causes according to quartiles of plasma vitamin D and time spent outdoors, and categories of sun sensitivity in PCOSun participants (n = 1119), stratified by PC aggressiveness.

Exposures	Non-aggressive^**†**^			Aggressive				
	Person years	Deaths^‡^ (n)	HR^††,‡^ (95% CI)	Person years	Deaths^‡^ (n)	HR^††,‡^ (95% CI)	Deaths^¥^ (n)	HR^††,¥^ (95% CI)
25 OHD (nmol/L)^*^								
14.23–53.82	934.8	21	1·00	1422.6	49	1·00	10	1·00
53·82–66·09	1050.7	20	0·98 (0·52, 1·84)	1187.5	28	0·70 (0·43, 1·13)	10	1·29 (0·52, 3.17)
66·09–80·25	996.7	18	0·92 (0·47, 1·80)	1034.0	19	0·55 (0·32, 0·95)	5	0·64 (0·21, 1·93)
80·25–174.68	994.9	23	0·90 (0·47, 1·72)	759.6	20	0·72 (0·41, 1·26)	5	1·04 (0·34, 3.20)
P heterogeneity			0·99			0·15		0·67
1,25 OHD (pmol/L)^*^								
4.84–67.24	881.4	20	1·00	1025.3	48	1·00	10	1·00
67.24–83·87	908.8	19	1·08 (0·57, 2·05)	1176.4	30	0·62 (0·39, 0.98)	12	1.08 (0·45, 2·57)
83·87–106·56	987.0	25	1·29 (0·70, 2·36)	1075.3	25	0·51 (0·31, 0·84)	NR^€^	0·47 (0·16, 1·44)
106·56–263.88	1200.0	18	0·73 (0·38, 1·40)	1126.7	13	0·28 (0·15, 0·52)	NR^€^	0·26 (0·07, 1.00)
P heterogeneity			0·34			0.00040		0.10
Time spent outdoors (hours)^*^								
0–10·5 (inclusive)	833.3	20	1·00	1022.9	39	1·00	NR^€^	1·00
10·5–18.0	948.0	20	0·89 (0·47, 1·67)	1100.3	29	0·70 (0·43, 1·14)	9	1·07 (0·40, 2·83)
18.1–28.0	1201.8	25	0·85 (0·47, 1·55)	1171.3	29	0·65 (0·39, 1·08)	9	1·15 (0·43, 3.09)
28.1–56·0	994.1	17	0·70 (0·35, 1·39)	1109.3	19	0·42 (0·24, 0·75)	NR^€^	0·48 (0·14, 1·64)
P heterogeneity			0·78			0·028		0·51
**Skin colour**								
Very fair	527.7	NR^€^	1·00	659.3	NR^6^	1·00	NR^€^	1·00
Fair	2264.9	45	0·72 (0·39, 1·33)	2506.6	68	1·01 (0·59, 1.73)	17	0·72 (0·27, 1.89)
Light olive	1084.9	22	0·68 (0·34, 1·39)	1087.0	28	1·07 (0·58, 1.95)	6	0·61 (0·19, 1·94)
Dark olive/Brown/Black	99.5	NR^€^	0·46 (0·06, 3.61)	150.8	NR^6^	0·54 (0·12, 2.37)	NR^€^	0·62 (0·07, 5.58)
P heterogeneity			0·67			0·84		0·86
**Ability to tan**								
Deeply tanned	1146.2	23	1·11 (0·65, 1·92)	1305.9	28	0·66 (0·42, 1·04)	NR^€^	0·45 (0·15, 1·38)
Moderately tanned	1869.2	32	1·00	2057.5	62	1·00	16	1·00
Mildly/no suntan	961.7	27	1·75 (1·02, 3·00)	1040.3	26	0·83 (0·52, 1·33)	NR^€^	1·55 (0·68, 3·54)
P heterogeneity			0·11			0·20		0·13

^*^Categories are quartiles of exposure.

^†^PC-specific mortality was not computable due to the small number of PC deaths in this category.

^**††**^Hazards ratio (HR) and 95% CI% CI adjusted for age, place of residence, socioeconomic status, region of birth, comorbidity, BMI, study, weekly outdoor exposure, season of blood collection and treatment (radical prostatectomy or other).

^**‡**^All-cause mortality.

^¥^PC-specific mortality.

^€^Not reported (NR) as sample size in one of these cells has less than 5 men.

In models with all variables as covariates, except vitamin D metabolite concentrations, (Table 4), mortality from all-causes was positively associated with age, treatment with androgen deprivation therapy, and Summer blood draw. Mortality from PC was positively associated with grade of disease at diagnosis- HR for aggressive PC relative to non-aggressive = 2·35 (95% CI: 1·17, 4·74, p-value 0·018).

**Table 4:** Adjusted mortality hazard ratios^1^ and 95% CI for deaths from all-causes and deaths from PC, according to PCOSun participant characteristics other than plasma vitamin D concentrations (n = 1119).

Participants’ characteristics	Person years	All-cause mortality		PC-specific mortality	
		Deaths (n)	HR^*^ (95% CI)	Deaths (n)	HR^*^ (95% CI)
**Age at diagnosis (years)**					
49–54	1161.9	15	1·00	5	1·00
55–59	2090.5	34	1·26 (0·68, 2.31)	9	0·95 (0·32, 2.85)
60–64	2420.6	47	1·36 (0·75, 2·46)	14	1.13 (0·40, 3.22)
65–85	2707.8	102	2·67 (1·52, 4.67)	13	0·97 (0·33, 2.85)
P heterogeneity			<0·0001		0·97
**Place of residence**					
Major cities	5690.4	115	1·00	30	1·00
Inner regional	2078.0	62	1·35 (0.96, 1·92)	NR^‡^	0·54 (0·23, 1·29)
Outer regional/Remote	612.4	21	1·36 (0·82, 2·26)	NR^‡^	0·92 (0·30, 2·79)
P heterogeneity			0·19		0·38
**Socioeconomic status (SES)**					
1 (Most advantaged)	2136.9	35	1·00	7	1·00
2	1428.6	32	1·26 (0·78, 2.06)	6	1·28 (0·42, 3.85)
3	2056.3	54	1·26 (0·79, 1·99)	12	1·85 (0·70, 4.93)
4	1581.7	47	1·33 (0·81, 2.16)	8	1·80 (0·61, 5.32)
5 (least Most advantaged)	1177.3	30	1·28 (0·75, 2.17)	8	2.39 (0·81, 7.06)
P heterogeneity			0·83		0·57
**Region of birth**					
Australia	6523.9	158	1·00	29	1·00
Other	1856.9	40	0.97 (0·68, 1·38)	12	1·33 (0·66, 2·68)
P heterogeneity			0·85		0·42
**Body Mass Index (BMI)**					
Under & Normal weight	2573.0	56	1·00	11	1·00
Overweight	4176.7	94	1·07 (0·76, 1·50)	19	1·02 (0·48, 2·17)
Obese	1631.1	48	1·46 (0·98, 2·19)	11	1·28 (0·53, 3·07)
P heterogeneity			0·14		0·82
**PC grade at diagnosis**					
Non-aggressive	3977.1	82	1·00	11	1·00
Aggressive	4403.7	116	1·30 (0·97, 1·73)	30	2·35 (1·17, 4·74)
P heterogeneity			0.075		0·018
**PC Treatment**					
Active Surveillance^†^	877.6	26	1·56 (0.98, 2·47)	NR^‡^	0.72 (0·16, 3·20)
Radical prostatectomy	5359.8	90	1·00	21	1·00
Low-dose radiation brachytherapy	299.2	5	1·15 (0·46, 2·87)	NR^‡^	NC^¥^
External beam radiotherapy	473.3	13	1·13 (0·62, 2·06)	NR^‡^	0·46 (0·06, 3.49)
Androgen deprivation therapy^††^	1370.9	64	2·17 (1·53, 3·06)	17	2.47 (1.23, 4.94)
P heterogeneity			0.0003		0·074
**Comorbidities**					
0	3290.1	65	1·00	14	1·00
1 or more	5090.7	133	1·05 (0·77, 1·44)	27	1·15 (0·58, 2.27)
P heterogeneity			0·75		0.68
**Season of blood draw**					
Spring	6195.8	138	1·00	28	1·00
Summer	1891.9	55	1·54 (1·12, 2.12)	NR^‡^	1·34 (0·66, 2·74)
Autumn&Winter	293.2	5	0.94 (0·38, 2·32)	NR^‡^	1.98 (0·45, 8.67)
P heterogeneity			0.029		0.53

^*^Hazards ratio (HR) and 95% CI adjusted for study, age, place of residence, SES, region of birth, BMI, PC treatment, PC grade at diagnosis, season of blood collection and comorbidities.

^†^Includes watchful waiting.

^††^Includes orchiectomy.

^‡^Not reported (NR) as sample size in one of these cells has less than 5 men.

^¥^Not computable (NC) as numbers of treatment-specific PC deaths are too small.

## Discussion

To the best of our knowledge, this is the first study to prospectively examine the association of post-treatment levels of plasma vitamin D metabolites with death after a PC diagnosis. We found no significant association between plasma 25(OH)D levels and risk of death after a diagnosis of PC. Risk of death from all-causes and from PC fell with increasing plasma 1,25(OH)_2_D levels. These trends appeared to be restricted to men with aggressive disease.

Previous epidemiological studies have focussed mainly on the effect of pre-diagnosis circulating 25(OH)D levels on mortality. They have consistently shown an inverse relationship between circulating pre-diagnosis 25(OH)D levels and all-cause mortality with mixed evidence for PC-specific mortality^6,7,9,20,21^. One study that measured post-treatment 25(OH)D levels, although small (37 participants with average time from treatment to blood collection 2·4 years), reported lower PC-specific mortality in men with 25(OH)D levels above 50 nmol/L in both pre- and post-treatment bloods, but a stronger association with post-treatment levels. These results suggest that time after treatment may be important to any association of circulating vitamin D with death in men with PC^10^. A combined analysis of the HPFS and the PHS showed no evidence of an association between 1,25(OH)_2_D levels measured before diagnosis and all-cause or PC-specific mortality^6^.

The differences between our results and those previously reported may be explained by the complex association between circulating 25(OH)D and 1,25(OH)_2_D. In young adults, the conversion of 25(OH)D to 1,25(OH)_2_D is tightly regulated, involving calcium and inorganic phosphate levels, as well as parathyroid (PTH) hormone actions. When circulating calcium levels are low, PTH levels are upregulated, which triggers the conversion of 25(OH)D to 1,25(OH)_2_D, which in turn promotes the intestinal absorption of calcium to restore calcium balance. Results from the Malmo Study in PC patients showed that the association between 25(OH)D levels and PC-specific mortality was modified by PTH and serum calcium levels^5^. Our inability to address effect modification by PTH or calcium, as we did not measure them, may explain some of the lack of association between 25(OH)D and PC-specific mortality in this study. Another factor to consider is the high 25(OH)D levels in our population compared to other studies. Over 90% of our participants had their bloods drawn in the warmer months (September to February) when ambient UV levels are high, and even those who had bloods drawn in autumn or winter had mean levels well within the sufficiency range (>70 nmol/L), probably due to outdoor sun exposure. The “warmer” months may raise men’s vitamin D levels to similar levels for all men, thereby possibly masking the effects of lower levels in the ‘cooler’ months on PC deaths.

Age of blood draw and timing of PC therapy may also play an important role in regulating vitamin D levels. Although tightly regulated in the young, seasonal fluctuations in 1,25(OH)_2_D levels have been reported in elderly people, possibly due to calcium dysregulation as a consequence of decline in bone mineral density^22–24^. Seasonal variation in 1,25(OH)_2_D levels was evident in PCOSun participants whose mean age at blood collection was 68 years. They were thus an older cohort than the Harvard cohorts (mean age 63 years in HPFS and 59 years in PHS)^6^. With regards to timing of blood collection relative to PC therapy, patients receiving androgen deprivation (ADT) and other adjuvant therapies are likely to experience disruption in their calcium homeostasis, which can result in loss of bone mineral density, and alter vitamin D status from the level it was before diagnosis to suit ‘current conditions’. The median time to blood collection was 5 years post-diagnosis in PCOSun, where a proportion of men had received primary treatment, whereas HPFS and PHS cohorts combined had blood specimens drawn at a median of 5 years before diagnosis. It may be that this difference in the sequence of blood draw and PC treatment partly explains our findings for an association between 1,25(OH)_2_D and PC mortality, that has not been observed in other studies.

That higher plasma 1,25(OH)_2_D levels might reduce PC-specific mortality in PC cases with aggressive disease was unexpected. Fang and co-workers from the HPFS and PHS reported a link between 25(OH)D deficiency and higher mortality only in men with aggressive PC, but they found no association with 1,25(OH)_2_D^6^. A similar analysis of Alpha-Tocopherol, Beta-Carotene Cancer Prevention Study reported increased PC-specific mortality in men with lower 25(OH)D, but no material difference in this association between men who had aggressive disease and those who did not^6,7^. The distinction between non-aggressive and aggressive PC is generally an intermediate to high risk of metastasis in the latter and a negligible to low risk of metastasis in the former^25,26^. The development of metastasis, which is usually to the bone, is led by the intercellular communication between PC cells and their microenvironment^11^. Both *in vivo* and *in vitro* studies have shown that the anti-proliferative effect of 1,25(OH)_2_D in influencing the interaction between tumour and its microenvironment is androgen-dependent and varies by disease aggressiveness^26^. Two clinical trial of cholecalciferol in PC patients showed a lowering in PSA levels, number of positive cores and cell proliferation in cases who received vitamin D supplementation^13,14^. They further reported associations between several tumour suppressive micro ribonucleic acids (miR-126-3p, miR-154-5p and miR-21-5p), an miR processing ribonuclease (DICER-1), and elevated levels of 1,25(OH)_2_D. This tumour suppressive miR and DICER-1 are known to be associated with reduced risk of PC recurrence thus suggesting that 1,25(OH)_2_D may alter the microenvironment to regulate the interaction between stroma and epithelium in prostate tissue in a pathway involving miRs^27,28^. This potential for 1,25(OH)_2_D to influence both intracellular and microenvironment activities may explain our finding for a significant effect of 1,25(OH)_2_D only in PC cases with aggressive disease.

In all the studies referred to above, the apparently beneficial effects of 25(OH)D were specific to PC death. In contrast, we observed evidence that 1,25(OH)_2_D protected against death from all causes. Levels 25OHD and 1,25(OH)_2_D are mainly regulated by PTH and calcium, with reducing dietary calcium lowering circulating 1,25D level, and increasing vitamin D intake and sun exposure increasing 1,25(OH)_2_D levels^29^. Few food products in Australia, however, naturally contain vitamin D or are fortified with it^30^, thus, it is unlikely that adequate intake of vitamin D is achievable here through diet modification alone, which then raises the potential for supplementation. Meta-analyses of vitamin D randomised trials reported a small reduction in total cancer mortality, but not incidence, in men and women supplemented with vitamin D (HR = 0·88; 95% CI: 0·78–0·98)^31,32^. Thus, an effect of high 1,25(OH)_2_D levels to reduce mortality from a number of cancers, including PC, is plausible, and consistent with other evidence of mortality benefits from vitamin D supplementation^31,32^. Two small clinical trials in PC patients showed the potential for vitamin D supplementation to reduce disease progression, but this may only have been achieved by high dose supplementation^13,14^. The duration of these trials however was too short to draw any conclusions on reducing mortality. Nonetheless, these studies raise the possibility that vitamin D-supplementation is beneficial in treating PC. There are currently a few clinical trials in progress that are exploring the long-term benefits of high dose vitamin D supplementation in PC patients^33,34^.

Our study has several strengths. Blood specimens were drawn well after the patients had completed primary treatment; the reasonably long follow-up thereafter reduced, but does not eliminate, the possibility of reverse causation explaining the apparent effects of 1,25(OH)_2_D on PC mortality. Most studies have used pre-diagnostic circulating vitamin D levels to predict PC risk. Low vitamin D levels, however, may be a consequence of the cancer, in that the cancer may cause lethargy leading to spending less time outdoors and consequently lowering vitamin D levels. Collecting blood some years after primary should have mitigated this effect. We used the National Comprehensive Cancer Network criteria for classifying PC as aggressive or not^18^. These criteria are consistent with how clinicians define aggressive disease and provide a comprehensive measure. The study’s main limitation is the small number of deaths from PC (n = 41), which limits the statistical power for detecting effects on prostate-specific mortality, and the lack of measurement of blood calcium and parathormone. Additionally, we chose not to adjust for multiple comparisons and, instead to evaluate the results in the context of prior evidence, biological plausibility, the number of tests performed, and the strengths of the observed associations, as recommended by several relevant experts^35–37^. While p-values are nominal for individual tests, the Type I error rate is likely to be inflated for the family of tests.

Our results suggest that high plasma 1,25(OH)_2_D after PC diagnosis and treatment may decrease all cause and PC-specific mortality, particularly in men with aggressive PC. With few known risk factors for PC identified to date, the results from this study suggests there may be prognostic value in post-diagnostic circulating levels of vitamin D for survival from aggressive PC, and possibly therapeutic value as well. These possibilities merit further research.

## Data Availability

Some access restrictions apply to the data underlying this study’s findings. The original human ethics committee approval for PCOS did not allow for data to be sent outside Australia. Furthermore, the PCOSun participants have not consented to their data being distributed beyond the PCOSun Investigators and their associates. Qualified researchers may submit a request to the corresponding author (visalinin@nswcc.org.au) and access will require additional ethics approval from the Cancer Council NSW HREC, including considerations of privacy for data sharing.
